# Hypothyroid offspring replacement with euthyroid wet nurses during lactation improves thyroid programming without modifying metabolic programming

**DOI:** 10.20945/2359-3997000000132

**Published:** 2019-04-26

**Authors:** Jorge Tapia-Martínez, Margarita Franco-Colín, Rocio Ortiz-Butron, Marisol Pineda-Reynoso, Edgar Cano-Europa

**Affiliations:** 1 Instituto Politécnico Nacional Departamento de Fisiología Escuela Nacional de Ciencias Biológicas Instituto Politécnico Nacional Ciudad de México México Departamento de Fisiología, Escuela Nacional de Ciencias Biológicas. Instituto Politécnico Nacional, Ciudad de México, México; 2 Instituto Politécnico Nacional Departamento de Fisiología Escuela Nacional de Ciencias Biológicas Instituto Politécnico Nacional Ciudad de México México Departamento de Fisiología, Escuela Nacional de Ciencias Biológicas. Instituto Politécnico Nacional, Ciudad de México, México; 3 Instituto Politécnico Nacional Departamento de Formación Básica Disciplinaria Escuela Superior de Medicina Instituto Politécnico Nacional Ciudad de México México Academía de Histología, Departamento de Formación Básica Disciplinaria, Escuela Superior de Medicina. Instituto Politécnico Nacional, Ciudad de México, México

**Keywords:** Congenital hypothyroidism, lactation, metabolic programming, thyroid programming, thyroid gland

## Abstract

**Objective:**

Determine the milk quality effect during lactation on the metabolic and thyroid programming of hypothyroid offspring.

**Materials and methods:**

Ten-week-old female Wistar rats were divided into two groups: euthyroid and thyroidectomy-caused hypothyroidism. The rats were matted and, one day after birth, the pups were divided into three groups: euthyroid offspring (EO), hypothyroid offspring (HO) and hypothyroid with a euthyroid replacement wet nurse (HRO). During lactation, the milk quality and offspring body length were evaluated. The body weight and energy intake were determined on a weekly basis, as well as the metabolic profile at the prepubertal (P35-36) and postpubertal (P55-56) ages. At P56, the animals were sacrificed, the adipose tissues were weighed and the thyroid glands were dissected for histological processing.

**Results:**

The milk of the hypothyroid wet nurse decreases proteins (16-26%), lipids (22-29%) and lactate (22-37%) with respect to euthyroid. The HO has a lower body weight gain (23-33%), length (11-13%) and energy intake (15-21%). In addition, HO presents impaired fasting glucose and dyslipidemia, as well as a reduction in seric thyroid hormone (18-34%), adipose reserves (26-68%) and thyroid gland weight (25-34%). The HO present thyroid gland cytoarchitecture alteration. The HRO develop the same metabolic alterations as the HO. However, the thyroid gland dysfunction was partially prevented because the HRO improved under about 10% of the serum thyroid hormone concentration, the thyroid gland weight although histological glandular changes presented.

**Conclusions:**

The replacement of hypothyroid offspring with a euthyroid wet nurse during lactation can improve the thyroid programming without modifying metabolic programming.

## INTRODUCTION

In recent years, several observations have assumed that metabolic diseases in adulthood emerge *in utero* or during lactation as a change result in the organs and systems under suboptimum conditions (1). In these periods, the neuronal and endocrine systems undergo critical periods of growth and maturation. They also develop their interrelationship signaling pathways (2). Thus, there are irreversible metabolic consequences when the gestation and/or lactation is/are perturbed. Several experiments in animal models have shown that nutrition (3), hormone-regulated metabolism (4,5), and other toxic influences such as thyroid disruptors affect metabolic and thyroid programming (6). In these conditions, adaptive metabolic strategies in the offspring change the metabolic set points in order to permit survival under critical conditions (7); for example, malnutrition in lactating rats was associated with changes in body weight (3,8) and thyroid dysfunction in their adult offspring (9).

Congenital hypothyroidism is a clinical and biochemical disease caused by low mother-transferred fetus thyroid hormones during gestation (10); it compromises hypothalamus-pituitary-thyroid gland (HPT) axis development and functionality (11,12). There is evidence that congenital hypothyroidism affects the offspring metabolic programming because it reduces growth hormone (13) and insulin-like growth factor binding protein (IGBFP)-2 responses (14). In addition, the wet nurse thyroid state is very important for the offspring metabolic and thyroid programming. It has been shown that thyroid hormones are necessary for preparing the breast for lactation (15) because they participate in the mammary gland lipolytic enzymes and proteases synthesis and expression. Thus, the milk of a hypothyroid wet nurse has low energetic metabolites, such as lipids and proteins (16,17); moreover, it contains a low hormone concentration (i.e., thyroid hormone IGF-1, growth hormone and leptin), which altered the metabolic actions in the offspring (17,18). In particular, thyroid hormones are important for the HPT development during the first ten days of lactation (19), and the adequate protein supply during lactation ensures the correct thyroid programming (20).

Altogether, congenital hypothyroidism causes metabolic programming changes. Further, the low quality of the milk produced by a hypothyroid wet nurse could modify thyroid gland programming. Therefore, the study aimed to prove that lactation in hypothyroid offspring with a euthyroid wet nurse, could improve hypothyroidism by causing metabolic and thyroid programming alteration.

## MATERIALS AND METHODS

### Animals and experimental design

All procedures were performed in accordance with the provisions of the laws and codes of Mexico in the seventh article of the General Health Law regarding health research (NOM-062-ZOO-2007). Additionally, the protocol was approved by the Internal Bioethical Committee (CEI-ENCB-021/2014).

Twenty female ten-week-old (250-280g) Wistar rats were used. The animals were housed in acrylic cages (20 × 30 × 18 cm) in a room with a light-dark cycle (12:12 h) and controlled temperature (21 ± 1 °C); food and water were offered *ad libitum*. The rats were conditioned for a week before starting the experiment; they were randomly divided into two groups: euthyroid wet nurse (EWN, n = 8) and hypothyroid wet nurse (HWN, n = 12). Thyroidectomy with parathyroid reimplantation was performed to the hypothyroid group. Thyroidectomy was carried out on rats anesthetized with ketamine (10 mg/kg, im)–xylazine (5 mg/kg, im). The previously described method was used (21). Briefly, by using a stereomicroscope (Zeiss, Germany) for better observation, the sternothyroid muscle was cut and the trachea was exposed. The parathyroid gland was located, dissected from the thyroid gland, and reimplanted into the surrounding neck muscle. The thyroid gland was carefully dissected to avoid injury to the laryngeal nerve and was completely excised. After surgery, enrofloxacin (10 mg/kg, im) and mefenamic acid (1 mg/kg ig) were administered over three days to alleviate pain and prevent infection. During surgery and in the recovery period, 0.05% of death was presented. Seven days post-surgery, three females were placed into a plastic cage with one male for mating. Only the thyroidectomized rats received pulses of 20 µg/kg sc T_4_ twice; first at the beginning of mating and, second, one day before labor; in previous experiments we found a low fertilization rate and a high delivery mortality rate of the HWN if these pulses of thyroid hormones were not administered. One day after birth, the pups were randomly assigned to groups (each group consisted of four males and four females raised by a wet nurse wet nurse) and were divided into three groups: euthyroid group (EO, n=12), hypothyroid group (HO, n=12) and hypothyroid replacement with euthyroid wet nurse (HRO, n=12). The HRO group consists of placing hypothyroid offspring with a euthyroid wet nurse.

### Metabolic evaluations

#### Energetic metabolites quantification in the nurses’ milk

The milk quality from each wet nurse in the different groups was tested. On day 7, 14 and 21 a dose of oxytocin (0.2 UI/kg i.p.) was administered to make the milk ejection process faster, then 10 min after oxytocin administration the rats were lightly anesthetized with ether and the mammary gland was gently pressed to simulate stimulating suction; the milk was then collected into individual vials (16). During the process the offspring were separated from the wet nurse for a period of 10 minutes to avoid stress by maternal separation. We obtained 500 µL stored at -20 °C. Until use, the amount of proteins, triglycerides, lactate and non-esterified fatty acid (NEFA) were quantified by using a kit from Randox.

#### Metabolic evaluations in offspring

During the whole treatment (P56), we measured the body weight and length two times a week during lactation. From P21 to P56, body weight and energy intake were determined weekly. We evaluated the rats at P35 and P55 because those dates are associated with prepubertal and postpubertal ages. After six hours of fasting, at P35 and P56 blood samples from the tail vein were obtained and the serum was separated and stored at −70 °C until assayed. Glucose, triglycerides, cholesterol and NEFA were measured. At P56, the animals were sacrificed by decapitation; the thyroid gland and mesenteric, retroperitoneal, epididymal or paraendometrial adipose tissue were dissected and were weighed immediately after removal.

## Thyroid programming in offspring

### Determination of serum concentrations of T3 and T4

We collected blood samples from the tail vein at 36 and 56 days post birth. For the determination of serum concentrations of T_3_ and T_4_ animals were not starved because the prolonged fast modifies the serum concentration of thyroid hormones. The samples were centrifuged at 3000 rpm for 5 min to obtain serum for thyroid hormone determination. We used the IMMULITE 2000 System (Siemens) to determine free T_3_ (Calibration Range: 40 to 600 ng/dL, Analytical Sensitivity: 19 ng/dL) and T_4_ (Calibration Range: 1.0 – 24 μg/dL, Analytical Sensitivity: 0.4 μg/dL).

### Morphological analysis of the thyroid gland

All animals were anesthetized at 56 days post birth with monosodium pentobarbital (45 mg/kg) after blood sampling. Six animals from each group underwent a total thyroidectomy using a stereoscopic microscope. We carefully preserved the thyroid gland lobes and the isthmus. The thyroid gland was immediately weighed on an analytical balance. Meanwhile, six animals from each group were used for histology. Briefly, 15 mm were dissected from the trachea containing the thyroid gland. It was fixed in 4% paraformaldehyde in PBS during 48 h. After that, the organs were processed by the conventional paraffin embedding technique. Serial sections of 7 µm were obtained and stained with hematoxylin-eosin.

## Statistical analysis

All of the variables evaluated were given as the mean ± standard error. The body weight, length, milk quantifications, clinical biochemistry of energetic metabolites and thyroid hormones were evaluated by repeated-measure two-way ANOVA and Student-Newman-Keuls *post hoc*. The two factors were age and thyroid state. The thyroid gland and adipose tissue weight were analyzed by one-way ANOVA and Student-Newman-Keuls post hoc. P < 0.05 was considered statistically significant.

## RESULTS

In Table 1, we observed the wet nurses milk quality during lactation. It was shown that hypothyroid wet nurses have a lower concentration of proteins (16-26%), triglycerides (22-29%), lactate (22-37%) and NEFA (10-11%) in all days that were evaluated in comparison to the euthyroid wet nurses.

**Table 1:** Milk quality evaluation during lactation

Milk energetic metabolites	Day lactation	Females	

		Euthyroid (EWN, n = 8)	Hypothyroid (HWN, n = 12)
Proteins (mg/dL)	7	53.23 ± 0.81^a^	45.04 ± 1.14^b^
	14	59.08 ± 1.48^a^	43.91 ± 0.984^b^
	21	57.73 ± 1.16^a^	47.26 ± 1.67^a^
Triglycerides (mg/dL)	7	163.06 ± 7.95^a^	128.33 ± 6.53^d^
	14	155.24 ± 9.59^b^	110.14 ± 7.63^a^
	21	153.13 ± 7.12^c^	119.75 ± 4.20^c^
Lactate (mg/dL)	7	7.90 ± 0.38^a^	6.13 ± 0.55^b^
	14	6.93 ± 0.57^b^	4.38 ± 0.475^d^
	21	9.77 ± 0.64^c^	7.03 ± 0.72^e^
NEFA (mmol/mL)	7	14.12 ± 0.79^a^	12.73 ± 0.65^b^
	14	13.48 ± 0.42^a^	12.03 ± 1.12^b^
	21	14.92 ± 0.39^a^	12.66 ± 1.54^b^

(a≠b≠c≠d≠e≠f) *p* < 0.05. RM two-way ANOVA and Student-Newman-Keuls *post hoc*.

**Figure 1:**
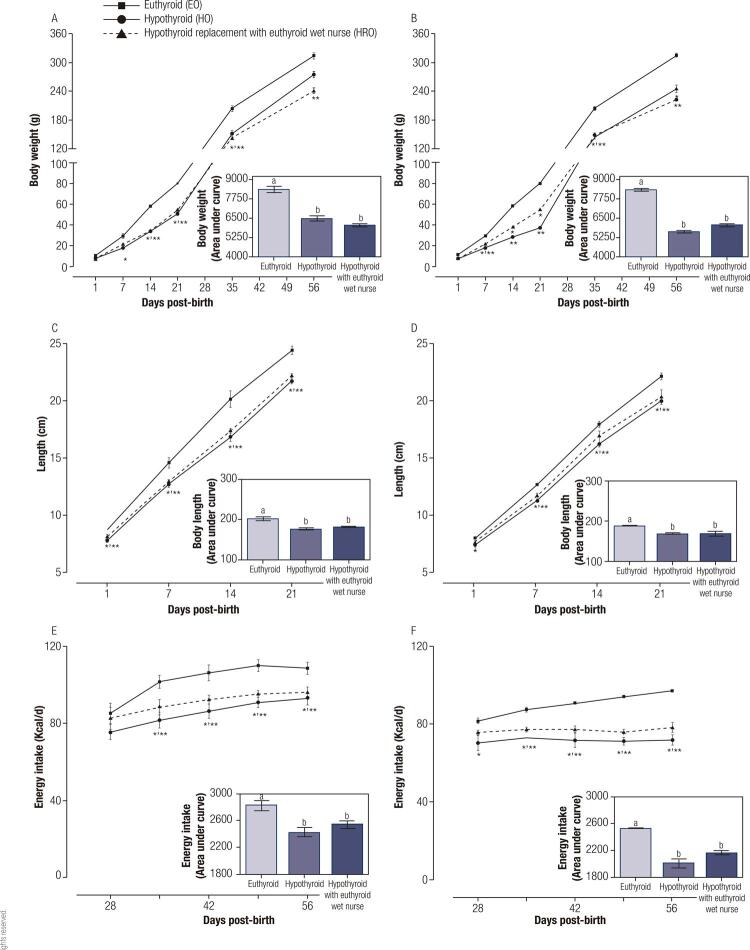
Effect of pre-natal and post-natal hypothyroid on body weight (A and B) and length (C and D) and energy intake (E and F) of male (A, C and E) and female (B, D and F) rats (n = 12). The data represent the mean ± the standard error, the bar graphs of the sides represent the area under the curve of the graph. (*) *p* < 0.05 vs the euthyroid group; (**) vs hypothyroid group at same day; a≠b *p* < 0.05. RM two-way ANOVA and Student Newman Keuls *post hoc*.

Figure 1 shows the hypothyroid metabolic effects on body weight (panel A and B), length (panel C and D) and energy intake (panel E and F). Perinatal and post-natal hypothyroidism reduces body weight gain, length and energy intake in male (body weight gain 23%, length 13%, energy intake 15% respect to AUC of EO) and female rats (body weight gain 33%, length 11%, energy intake 21% respect to AUC of EO). Meanwhile, the hypothyroid group with a euthyroid wet nurse has the same metabolic pattern as the hypothyroid group (male body weight gain 28%, length 11%, energy intake 11%, female body weight gain 28%, length 18%, energy intake 15%, respect to AUC of EO).

The clinical biochemistry of the energetic metabolites that were analyzed is shown in Table 2. The hypothyroid and hypothyroid with euthyroid wet nurse groups suggested increased glucose (12-25%), triglycerides (47-67%) and cholesterol (55-62%) and reduced NEFA (17-28%) in both ages and genders.

**Table 2:** Effect of pre-natal and post-natal hypothyroidism in clinical biochemistry of energetic metabolites in males and females at the pre-pubertal and post-pubertal age

Serum concentration		Prepubertal age (P35)			Postpubertal age (P55)		
	
		Euthyroid (EO; n = 12)	Hypothyroid (HO; n = 12)	Hypothyroid with Euthyroid wet nurse (HRO; n = 12)	Euthyroid (EO; n = 12)	Hypothyroid (HO; n = 12)	Hypothyroid with Euthyroid wet nurse (HRO; n = 12)
Male	Glucose (mg/dL)	95.60 ± 1.25^a^	117.00 ± 2.72^b^	110.60 ± 4.65^b^	95.40 ± 0.872^a^	119.40 ± 4.37^b^	107.80 ± 4.02^b^
	Triglycerides (mg/dL)	78.80 ± 3.61^a^	120.00 ± 6.74^b^	116. 60 ± 7.45^b^	79.00 ± 3.78^a^	132.20 ± 9.49^b^	130.60 ± 7.22^b^
	Cholesterol (mg/dL)	50.00 ± 1.92^a^	80.00 ± 2.45^b^	77.60 ± 2.79^b^	56.80 ± 3.18^a^	91.60 ± 3.59^b^	89.80 ± 1.50^b^
	NEFA (mmol/ mL)	1.36 ± 0.07^a^	1.08 ± 0.06^b^	0.98 ± 0.05^b^	1.74 ± 0.11^a^	0.96 ± 0.05^b^	0.96 ± 0.19^b^
Female	Glucose (mg/dL)	95.20 ± 1.16^a^	114.80 ± 4.26^b^	104.80 ± 4.64^b^	94.40 ± 1.81^a^	112.80 ± 6.65^b^	108.00 ± 4.50^b^
	Triglycerides (mg/dL)	85.60 ± 4.23^a^	117.20 ± 7.46^b^	128.40 ± 9.96^b^	87.80 ± 3.15^a^	130.40 ± 9.84^b^	133.00 ± 10.38^b^
	Cholesterol (mg/dL)	57.20 ± 3.41^a^	88.00 ± 3.44^b^	86.20 ± 3.31^b^	57.40 ± 2.23^a^	90.80 ± 2.52^b^	89.40 ± 1.47^b^
	NEFA (mmol/ mL)	1.34 ± 0.06^a^	1.15 ± 0.06^b^	1.01 ± 0.05^b^	1.65 ± 0.09^a^	0.88 ± 0.06^b^	0.83 ± 0.14^b^

(a≠b) *p* < 0.05. RM two-way ANOVA and Student-Newman-Keuls *post hoc*.

**Figure 2:**
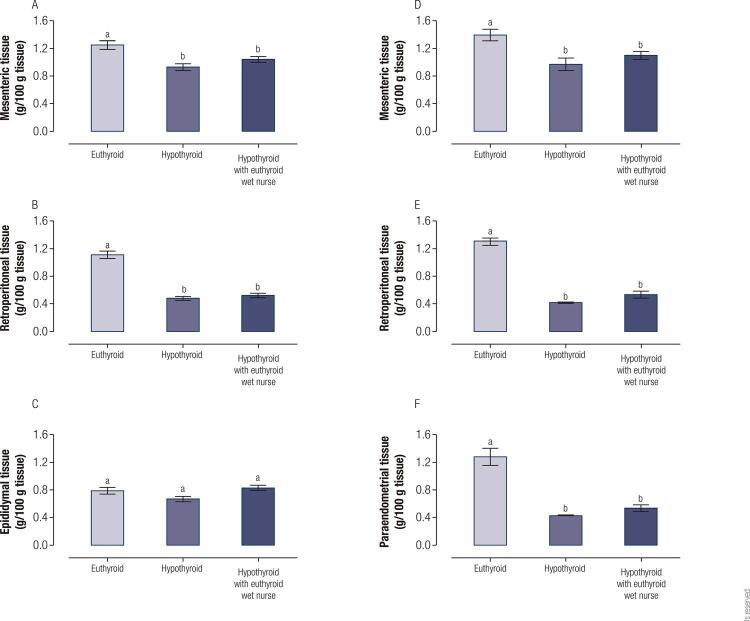
Effect of pre-natal and post-natal hypothyroidism on reserves adipose tissue quantification of males [(A) mesenteric, (B) retroperitoneal, (C) epididymal] and females [(D) mesenteric, (E) retroperitoneal, (F) paraendometrial] (n = 12). (*) *p* < 0.05 vs the euthyroid group; a≠b *p* < 0.05. RM one-way ANOVA and Student Newmann Keuls *post hoc*.

Figure 2 shows the reserves of adipose tissue on P56. We found that females in the HO and HRO groups reduced all reserves of adipose tissue (mesenteric 31%, retroperitoneal 68%, paraendometrial 67% for HO and mesenteric 21%, retroperitoneal 59%, paraendometrial 58% for HRO with respect to EO). Meanwhile, males of the same groups reduced only mesenteric (26% for HO and 17% for HRO respect to EO) and retroperitoneal (57% for HO and 53% for HRO respect to EO) adipose tissue.

The thyroid hormone concentrations and thyroid weight glands are shown in Table 3. It was observed that pre-natal and post-natal hypothyroidism caused a reduction on T_3_ and T_4_ at P35 (male reductions: T_3_ 19% and T_4_ 20%; female reductions: T_3_ 22% and T_4_ 18%) and P56 (male reductions: T_3_ 22% and T_4_ 34%; female reductions: T_3_ 22% and T_4_ 25%) in both genders. However, the hypothyroid group that had a euthyroid wet nurse presented a lower thyroid hormone reduction than the hypothyroid group at P35 (male reduction: T_3_ 3% and T_4_ 8%; female reduction: T_3_ 7% and T_4_ 6%) and P56 (male reduction: T_3_ 14% and T_4_ 19%; female reduction: T_3_ 11% and T_4_ 7%). Also, males and females of the hypothyroid group had a lower weight of the thyroid gland (34% for males and 25% for females).

**Table 3:** Effect of pre-natal and post-natal hypothyroidism on serum thyroid hormones quantification and the thyroid gland weight of males and females at the pre-pubertal and post-pubertal age

Serum concentration		Prepubertal age (P36)			Postpubertal age (P56)		
	
		Euthyroid (EO)	Hypothyroid (HO)	Hypothyroid with Euthyroid wet nurse (HRO)	Euthyroid (EO)	Hypothyroid (HO)	Hypothyroid with Euthyroid wet nurse (HRO)
Male	T_3_ (ng/dL) (n = 12)	104.34 ± 5.37^a^	83.78 ± 3.89^b^	96.46 ± 1.68^c^	126.80 ± 4.62^a^	84.58 ± 3.83^b^	103. 02 ± 2.26^c^
	T_4_ (µg/dL) (n = 12)	9.20 ± 0.14^a^	7.50 ± 0.08^b^	8.96 ± 0.20^c^	10.92 ± 0.22^a^	8.58 ± 0.27^b^	9.40 ± 0.12^c^
	Thyroid gland weight (mg) (n = 6)	-----	-----	-----	18.20 ± 1.21^a^	12.10 ± 0.48^b^	14.94 ± 1.146^c^
Female	T_3_ (ng/dL) (n = 12)	88.66 ± 2.69^a^	73.36 ± 2.18^b^	94.34 ± 2.61^c^	124.26 ± 8.96^a^	94.16 ± 5.22^b^	90.78 ± 2.52^c^
	T_4_ (µg/dL) (n = 12)	10.22 ± 0.21^a^	7.98 ± 0.26^b^	9.54 ± 0.16^c^	10.60 ± 0.22^a^	8.32 ± 0.19^b^	9. 00 ± 0.17^c^
	Thyroid gland weight (mg) (n = 6)	-----	-----	-----	17.2 ± 1.02^a^	12.94 ± 0.37^b^	15.92 ± 0.36^c^

(a≠b≠c) *p* < 0.05. RM two-way ANOVA and Student-Newman-Keuls *post hoc*. (---- not evaluated).

**Figure 3:**
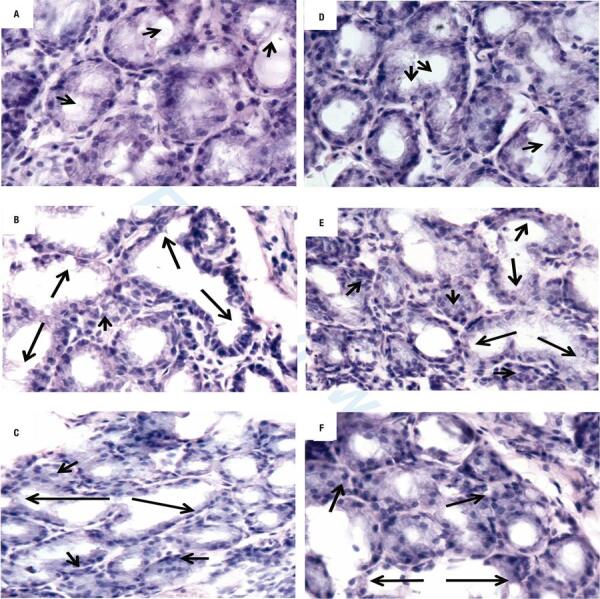
Effect of pre-natal and post-natal hypothyroidism on thyroid gland cytoarchitecture. The photomicrographs show histological cuts with HE stain. Euthyroid males (A) and females (D); hypothyroid males (B) and females (E); hypothyroid replacement with euthyroid wet nurse males (C) and females (F). Arrows show the follicular organization, colloid and reabsorption lacunae.

The arrows in Figure 3 show in the male (A) and female group (D) of EO, the thyroid gland has hemispherical follicles with follicular proper organization, abundant colloid and reabsorption lacunae are observed. In the male (B) and female (E) of HO, the gland has the presence of larger follicles without the hemispherical characteristic shape with little colloid; in addition, central areas with follicular disorganization were observed. With respect to the HRO male (C) and female (F) groups, the follicles have hemispherical morphology, presenting abundant larger follicles without colloid and flat cells, as well as areas with follicular disorganization.

## DISCUSSION

Lactation is a critical period because important cognitive and metabolic events occur (22). Adverse changes in the environment, such as malnutrition and abnormal weight-regulatory hormonal concentration in lactating rats, are associated with metabolic disease in adulthood (23). Thyroid hormones (TH) are important in the mammary gland function because they participate in the adequate initiation and progress of lactation (15). In addition, the TH regulates energetic metabolic pathways as carbohydrates, lipids and proteins. Thus, the thyroid state in the wet nurses modifies the milk quality and the metabolic programming in the offspring (17). However, research has not yet determined whether the metabolic and thyroid programming alterations in hypothyroid offspring (HO) are the result of low nutritional intake of proteins and lipids and/or the low thyroid hormone contribution from the wet nurse during lactation. Thus, the aim of this study was to determine if hypothyroid offspring replacement with euthyroid wet nurses (HRO) during lactation develops thyroid and metabolic programming alterations during pre-pubertal and post-pubertal ages.

We observed that hypothyroid wet nurses (HWN) produce milk with lower energetic metabolites (proteins and lipids) than that of euthyroid wet nurses (EWN). Hypothyroidism affects mammary function and lactation because it reduces the activity and expression of enzymes involved in energetic metabolic pathways as lipogenesis, lipolytic and protein synthesis. Thus, the HWN compromise the offspring nutritional state by causing nutrient restriction during lactation and metabolic readjustment (24). We observed that HO has metabolic alterations and thyroid dysfunction in females and males. There are hormonal inputs that are related to the metabolic programming such as glucocorticoid, insulin and leptin (25). However, in recent years the relationship between TH and metabolic programming has been studied because TH can probably participate in metabolic programming during critical stages of development (26).

In mammals, many neuroendocrine networks that are responsible for metabolic and thyroid programming become organized during the intrauterine and perinatal stages of development. During puberty a complex developmental event of continuum changes occurs, leading a sexual and somatic maturation of the individual (27). However, nutrimental restriction during pregnancy and lactation may induce changes within the programming that affects the developmental process of puberty and the presence of metabolic and thyroid disorders that are exacerbated in the long term (28). Even though the mechanism of this effect is still unknown, metabolic programming may be affected permanently by an imbalance in the TH supply during fetal life as it occurs in congenital hypothyroidism and causes long-term changes in the offspring. It has been observed that congenital hypothyroidism decreased insulin secretion in adulthood (29,30), which is related to high glucose concentration (31). In addition, lactation is an important period in which the correct metabolic and thyroid programming is established (23). It is possible that, by restoring lactation on HO, metabolic and thyroid programming could be improved. To analyze it, we employed the HRO to restore the hormonal and nutrimental supply during lactation. It has been shown that cross fostering during the first postnatal days can be an effective method to improve the newborn nutritional status in some species such as pigs (32). However, in rats, cross fostering is usually used to study behavior and thyroid disruptors in relationship with some metabolic parameters (33). So, this is the first study to use cross fostering in rats during lactation through the HRO with a EWN. However, this methodological strategy does not prevent metabolic programming; nevertheless, the thyroid programming was mildly improved. It has been observed that HWN reduces IGF-I and TH concentration in the milk. Thus, the metabolic restoration during lactation with EWN through quality milk provides the correct milk metabolic hormones concentration, but it does not prevent hypothyroidism. On other hand, the euthyroid milk provides more energetic metabolites, such as lipid and proteins, than hypothyroid milk (17). Perhaps the metabolic programming was not improved in HRO because the congenital hypothyroidism causes metabolic programming alteration. Furthermore, regarding thyroid programming, in the HRO, thyroid gland dysfunction was partially prevented. It has been observed that maternal TH during the end of gestation and throughout the ten lactation days programmed the HPT in the offspring (19,34). In addition, the nutritional supply during lactation it is important for the normal thyroid function (3). Thus, in our model, when the HO was fed with the euthyroid milk, the energetic metabolites and the thyroid hormone improve the HPT axis.

In conclusion, we suggest that the thyroid status of the mother during gestation stage is very important for the correct metabolic programming in the offspring. Moreover, lactation plays an important role in thyroid programming.
